# Pulmonary Combined Large Cell Neuroendocrine Carcinoma

**DOI:** 10.3389/pore.2022.1610747

**Published:** 2022-11-23

**Authors:** Meihui Li, Lan Yang, Hongyang Lu

**Affiliations:** ^1^ Zhejiang Key Laboratory of Diagnosis & Treatment Technology on Thoracic Oncology (Lung and Esophagus), Cancer Hospital of the University of Chinese Academy of Sciences (Zhejiang Cancer Hospital), Hangzhou, China; ^2^ Department of Thoracic Medical Oncology, Cancer Hospital of the University of Chinese Academy of Sciences (Zhejiang Cancer Hospital), Hangzhou, China; ^3^ Institute of Basic Medicine and Cancer (IBMC), Chinese Academy of Sciences, Beijing, China; ^4^ The First Clinical Medical College, Wenzhou Medical University, Wenzhou, China

**Keywords:** pulmonary combined large cell neuroendocrine carcinoma (CLCNEC), pathological characteristics, diagnosis, therapy, targeted therapy

## Abstract

Pulmonary combined large-cell neuroendocrine carcinoma (CLCNEC) is a rare neuroendocrine tumor pertained to lung large cell neuroendocrine carcinoma (LCNEC) with aggressive behavior and poor prognosis generally. The clinical features of CLCNEC are not specific including cough, expectoration, chest distress, chest pain, etc., which are prone to have different manifestations of the mixed components. Owing to the low incidence, there are few related small-scale retrospective studies and case reports. Currently, the treatment regimen of CLCNEC mainly refers to LCNEC that complete surgical resection is preferred in the early stage and according to previous researches, platinum-based small cell lung cancer (SCLC) standard treatment regimen showed promising results in postoperative and advanced CLCNEC as compared to that of non-small cell lung cancer (NSCLC). Adenocarcinoma-CLCNEC more likely harbor driver gene mutation, and may benefit from targeted therapy. As for immunotherapy, more clinical trial data are needed to support its benefits. This article will fill the gap and will provide new insight into the clinical characteristics, pathological diagnosis and treatment endeavors of CLCNEC.

## Introduction

Originating from argyrophilic cells in the mucosa of lung and bronchus [1, 2], lung large cell neuroendocrine carcinoma (LCNEC) is a relatively rare carcinoma representing 3% of all lung cancers [3] and 2.1%∼3.5% of pulmonary surgically resected specimens [4, 5]. International Agency for Research on Cancer (IARC) classification of Thoracic Tumors (5th Edition) published in 2021 classified pulmonary combined large cell neuroendocrine carcinoma (CLCNEC) as a subtype of LCNEC associated with LCNEC components and epithelial components such as adenocarcinoma or squamous carcinoma [6]. The CLCNEC portend poor prognosis upon comparison with LCNEC, with greater extend of lymph nodes and distant metastasis, and are characterized by the clinical stages III∼IV [7]. So far, the CLCNEC is mainly treated with complete surgical resection in early stages of I and II, and the advanced stages are treated with chemotherapy to relieve symptoms, which is similar to the treatment endeavors of LCNEC. Owing to the rarity of CLCNEC, this article intends to summarize the clinical manifestations, pathological features, treatment options and prognosis of CLCNEC depending on a number of domestic and foreign literature and will serve as a blueprint for accurate diagnosis and treatment and improved prognosis.

## Clinical Characteristics

In 2015, world health organization (WHO) grouped typical carcinoid (TC), atypical carcinoid (AC), small cell lung cancer (SCLC) and LCNEC into neuroendocrine tumors (NETs) [8] and classfied CLCNEC into a rare subtype of LCNEC. CLCNEC exhibited morphological and immunohistochemical features of LCNEC in some regions, and that of NSCLC (adenocarcinoma, squamous cell carcinoma, spindle cell carcinoma, giant cell carcinoma, etc.) components in other regions [9,10,11]. Adenocarcinoma is the most common component in CLCNEC accounting for around 70% [12, 13]. In addition, LCNEC combined with SCLC part was classified as a type of combined SCLC [14]. More than 10% of LCNEC patients were diagnosed with CLCNEC before the first treatment [15, 16].

Instead of focusing on specified analysis of pure or combined parts, current studies on CLCNEC generally take all components as a whole [17, 18]. By far, there are only a few related case reports and small-scale retrospective studies reported on CLCNEC which could not provide sufficient evidences about the clinical outcome. The incidence of CLCNEC is found associated with male sex, median-older age and heavy smoking [17] which was proved that there’s no significant difference in gender, age and serum tumor markers between CLCNEC and pure LCNEC [5, 19]. Generally the initial stage is undiagnosed frequently because patients are more likely to present nonspecific symptoms, such as cough, expectoration, chest tightness, chest pain, dyspnea, and hemoptysis resulting in a high lymph node metastasis rate (60%–80%), high distant metastasis rate (40%), and a high degree of malignancy at the time of diagnosis [20]. Based on several domestic and foreign case reports and small retrospective studies [12,13,21–26], CLCNEC and LCNEC showed similarities in clinical symptoms and tumor aggressiveness. Handa Y et al. [21] collected 64 LCNEC patients with complete resection history containing 33 cases of CLCNEC and 31 cases of pure LCNEC. The study found that two subtypes of LCNEC exhibit almost same percentage of pleural/lymphatic/vascular invasion and distant recurrence (more than 80% and 40% respectively). Yang T et al. [22] gathered a cohort of 96 CLCNEC patients, including 71 cases of adenocarcinoma-LCNEC and 25 cases of squamous-LCNEC. The authors raised the possibility of clinical manifestations of CLCNEC to be influenced by mixed components: adenocarcinomas-LCNEC is more common in young female never-smokers, and the lesions are often peripherally located with driver gene mutation; while squamous-LCNEC was more common in elder male patients (over 65 years of age) and was more likely to be centrally located. But there was no significant distinction in disease free survival (DFS) and overall survival (OS) between the two CLCNEC subtypes. Moreover, the age and location of onset seem to be more correlated with the NSCLC part. The clinical characteristics and treatment options of patients are gathered in Table 1.

**TABLE 1:** Clinical characteristics and therapy of CLCNEC patients.

References	Sex	Age	Smoking	Combined component	Therapy
[12]	Male 28	<61: 11	Yes: 26	AD: 21	un
	Female 2	≥61: 19	Never: 4	SCC: 9	un
[13]	Male 96	<65: 67	Yes: 64	AD: 82	S + PAC: 88
	Female 20	≥65: 49	Never: 52	SCC: 34	S + R: 18
[25]	Male	77	yes	SCC	S + PAC
[29]	Male	73	yes	AD + SA	C + I
[31]	Female	61	never	AD	C + T
[32]	Female	57	yes	AD	T
[45]	Male	54	yes	AD	S + PAC + I
[46]	Male	66	Un	AD	S
[47]	Male	46	Un	SCC	S + PAC

Abbreviations: AD, adenocarcinoma; SCC, squamous cell carcinoma; SA, sarcomatoid; un:unknown; S, surgery; PAC, postoperative adjuvant chemotherapy; C, chemotherapy; R, radiotherapy; I, immunotherapy; T, targeted therapy.

## Diagnosis and Pathological Features

The diagnosis of CLCNEC is divided into two parts. Pathological manifestations of LCNEC part are complicated and volatile. The observation of the cell and tissue morphology through light microscopy combined with immunohistochemical (IHC) features and neuroendocrine particles under electron microscopy are required for precise and accurate diagnosis [27]. The pathological diagnostic criterion for LCNEC classified by WHO in 2021 are as follows: 1) neuroendocrine morphology; 2) high cell division ratio; 3) abundant necrotic tissue; 4) cytological characteristics of NSCLC; 5) immunohistochemistry: one or more neuroendocrine markers were stained or electron microscopy showed neuroendocrine granules. Previous researches [13,22–25] found that the arrangement of tumor cells in the part of CLCNEC was generally consistent with LCNEC, revealing palisade or chrysanthemum cluster arrangement, often with prominent nucleoli and multiple nuclear divisions (usually 30-100/10 HPF). Chromogranin A (CgA), Synaptophysin (Syn) and neural cell adhesion molecule 56 (CD56) were diffusely and strongly expressed in LCNEC part. While there were focally adenocarcinoma, squamous cell carcinoma and spindle cell carcinoma components which expressed the corresponding epithelial-derived IHC markers. For example, adenocarcinoma expresses NapsinA, while squamous cell carcinoma expresses CK5/6, p40 and p63. The immunohistochemical composition of spindle cell carcinoma is complex, requiring further judgment [9, 28]. Adenocarcinoma is the most common mixed component of CLCNEC accounting for around 70%, followed by squamous cell carcinoma representing 20% of CLCNEC [12, 13]. Sarcomatoid was reported as a combined component in a case report recently [29].

Ando T et al.[30] analyzed a CLCNEC with mixed adenocarcinoma subtypes including papillary, acinar and lepidic. The next-generation sequencing (NGS) results of each component suggested that trans-differentiation occurred in a single tumor through the accumulation of gene mutations by combined DNA and RNA analysis. Mutations in epidermal growth factor receptor (EGFR) and associated rho guanine nucleotide exchange factor (ARHGEF12) were detected as trunk mutations, common among the four lesions, indicating each subtype had the same clonal origin. While, gene mutations including PTEN, MST1R, and PIK3CA were noted during transdifferentiation from acinar adenocarcinoma to LCNEC. Miyoshi T et al. [15] reported 10 cases of CLCNEC harboring driver gene mutations analyzed by NGS, 5 of which harbored the same mutations in the two tumor components including high frequency TP53 and RB1 inactivation. The remaining 5 cases exhibited mutations in: EGFR, insulin-like growth factor receptor (IGF1R), cellular-mesenchymal to epithelial transition factor (c-MET), PIK3CA, and Kirsten rat’s arcomaviral oncogene (KRAS) in NSCLC part. Lim CA [31] reported that ALK IHC showed strong cytoplasmic staining of tumor cells in both LCNEC and adenocarcinoma components. FISH studies were performed, and two morphologies were scored separately. Signal patterns of both were similar, and consistent with an ALK rearrangement. Sakamoto T [32] proposed that BRAF V600E was tested positive by immunostaining (anti-BRAF V600E rabbit monoclonal antibody clones) and NGS in both adenocarcinoma and LCNEC part. Therefore, it’s possible that LCNEC and mixed components may share the same mutations but developed towards different morphology or performed subtype-transformation, which needs further exploration. Overview of individual mutations in each component of LCNECs combined with another cell type are gathered in Table 2.

**TABLE 2:** Overview of individual mutations in each component of LCNECs combined with another cell type.

Mutation	Case 1–10 [13]	Case 11–17 [13]	Case 18 [13]	Case 19 [15]		Case 20 [15]		Case 21 [15]		Case 22 [15]		Case 23 [15]		Case 24 [15]		Case 25 [15]		Case 26 [15]		Case 27 [15]		Case 28 [15]		Case 29 [31]		Case 30 [32]		Case 31 [43]		Case 32 [44]		Case 33 [45]	
				L	A	L	A	L	A	L	A	L	A	L	S	L	S	L	S	L	S	L	S	L	A	L	A	L	A	L	A	L	A
TP53				H214R	H214R	E258G	E258G			K120E		283P				R158L		N131Y	N131Y	E285		V157F	V157F										
RB1								W99						R579	R579																		
PIK3CA																	E545K																
KRAS											G12V																						
EGFR	19 del*	21 L858R*	20 ins*						E746_A750del																								
IGF1R													G870																				
MET																					V1088M												
ALK																								Re	Re			Re		Re		Re	
BRAF																										V600E	V600E						
NM																													√		√		√

Abbreviations: L, LCNEC component; A, adenocarcinoma component; S, squamous cell carcinoma component; Re, rearrangement; NM, not mentioned; del, deletion; ins, insertion.

*The mutation coming from which component is not mentioned in the article.

## Chemotherapy

Currently, the common treatment regimen of CLCNEC mainly refers to LCNEC that complete surgical resection is preferred for limited-stage (I-IIIA) according to the NSCLC guidelines announced by The National Comprehensive Cancer Network (NCCN). The SCLC-like or NSCLC-like postoperative adjuvant chemotherapy selection for advanced or metastatic CLCNEC is remained controversial. Recent clinical studies reported the prognosis of LCNEC patients receiving SCLC chemotherapy regimen was more effective as compare to that of NSCLC [33–36]. Zhang JT et al. [12] denied the aforementioned similar results on the basis of low-level evidences. A cohort of 381 LCNEC patients including 30 cases of CLCNEC were retrospectively evaluated for adjuvant treatment and the first-line treatment based on their treatment course. In the adjuvant group, median DFS was non-significantly longer for SCLC-based regimens than for NSCLC-based regimens (*p* = 0.112). The first-line group exhibit significantly longer median progression-free survival (PFS) for SCLC-based regimens than for NSCLC-based regimens (11.5 vs. 7.2 months, *p* = 0.003). Additionally, median OS (mOS) was non-significantly shorter for CLCNEC than pure LCNEC (*p* = 0.083) due to limited sample size and potential follow-up bias. This result point out SCLC regimen as more appropriate choice as either first-line or adjuvant chemotherapy, when compared to the NSCLC regimen for LCNEC treatment. CLCNEC should be managed in a multidisciplinary setting, confirming the adjuvant chemotherapy (especially the SCLC regimen) paramount importance to improve patients’ outcome [37].

## Potential Future Opportunities

### Immunotherapy

In the last decade, immunotherapy has dramatically changed the natural history of NSCLC optimizing OS and life quality of patients [38]. Preliminary data have suggested that response rates of CLCNEC to immune checkpoint inhibitors (ICIs) were perhaps above what might have been expected for a low PD-L1 cancer, particularly in aggressive/advanced diseases [39, 40]. Xu J et al. [41] reported a case of CLCNEC who underwent adjuvant chemotherapy, radiotherapy and maintenance therapy with durvalumab. The above treatment regimen for the CLCNEC brought an evaluation of complete remission (CR). A 73-year-old male with CLCNEC of the lung containing adenocarcinoma and sarcomatoid components was treated with chemotherapy consisting of carboplatin and nanoparticle albumin-bound (nab)-paclitaxel plus atezolizumab, which was decided in accordance with the histological evaluation of the components. This treatment resulted in partial response (PR) and remained durable for 12 months [29]. The aforementioned finding indicated that some CLCNEC patients may benefit from immunotherapy due to high TMB, but more clinical data are needed to support this statement and can assist in highlighting the standardized treatment method for CLCNEC.

### Targeted Therapy

CLCNEC presented a higher probability of driver gene mutations than pure LCNEC due to its NSCLC part [15, 42]. Wang Y et al. [13] reported a retrospective study of 70 CLCNEC patients who underwent adjuvant chemotherapy after surgery analyzed by NGS. 18 patients with EGFR mutations of CLCNEC were observed, including 10 patients with 19 exon deletions mutation, 7 patients with single L858R mutation in exon 21, and 1 patient with exon 20 insertion mutation. A total of 9 CLCNEC patients who developed distant metastases after surgery were treated with tyrosine kinase inhibitor (TKI). Among them, 4 patients harboring anaplastic lymphoma kinase (ALK) mutation received crizotinib, and 5 patients with EGFR 19del/L858R mutations received either first-generation TKI (gefitinib, icotinib, or erlotinib) or second-generation TKI (afatinib). The objective response rate (ORR) of 9 patients was 66.7%. Yang Z et al. [22] analyzed 60 CLCNEC resected samples using NGS, and found that 23 patients with positive results were all adenocarcinoma-CLCNEC, including 17 cases of EGFR mutation, 4 cases of ALK rearrangement and 2 cases of KRAS mutation. In the back line, 4 patients with ALK rearrangement were treated with crizotinib, and 5 patients with EGFR mutation were treated with either first-generation TKI (including gefitinib, ectinib, and erlotinib) or second-generation TKI (afatinib). Several case reports have detected ALK fusion from CLCNEC patients who benefited from alectinib or crizotinib [31,43–45]. The above data point out targeted therapy as the most feasible treatment option for CLCNEC patients diagnosed with mixed adenocarcinoma components.

## Prognosis

CLCNEC exhibit a high degree of malignancy with relatively poor prognosis. Wang Y et al. [13] conducted postoperative study treating a cohort of 116 CLCNEC patients. In which 51 patients received NSCLC standard treatment regimen, and 37 patients received SCLC treatment regimen. The researchers revealed tumor size, pN stage, peripheral CEA level, and adjuvant chemotherapy as independent prognostic parameters for DFS and OS in CLCNEC patients. J. T. Zhang et al. [12] recruited 30 CLCNEC patients, and multivariable Cox regression analysis denied the prediction of IHC marker poorer OS with elevated NSE level, other than TNM stage (Tumor, Node, Metastasis) (*p* < 0.001). Comparing mOS of pure LCNEC with CLCNEC, the latter was found more aggressive (*p* = 0.083). Handa Y et al. [21] collected 33 cases of CLCNEC patients and 31 of pure LCNEC. The multivariate Cox regression analysis found that vascular invasion (HR = 2.77; 95% CI: 1.09–9.98; *p* = 0.020) and pathological stage (HR = 2.34; 95% CI: 1.25–10.55; *p* = 0.029) to be independent prognostic factors of OS. There is no statistical difference in 5-year OS rates and 5-year recurrence-free survival (RFS) rates of CLCNEC and pure LCNEC (61.8% vs. 52.2%, *p* = 0.82; 42.4% vs. 43.9%, *p* = 0.96). Therefore, the researchers concluded that the prognosis of CLCNEC patients were equivalent to that of pure LCNEC. The differences in OS between CLCNEC and LCNEC patients in the above literatures may be caused due the limited data availability in retrospective studies with selective bias.

## Conclusion & Prospect

A growing evidences proved that CLCNEC is a neuroendocrine carcinoma with high-grade morphological and biological heterogeneity and aggressive tumor malignancy. Due to lack of definitive evidences, the CLCNEC is short of standardized treatment strategy and follow LCNEC diagnosis and treatment strategies more evidently the complete surgical resection is preferred in early stages of I and II. In postoperative and advanced stages, SCLC-like standard chemotherapy is advised to be the best option as compared with NSCLC chemotherapy. The CLCNEC patients rarely benefit from immunotherapy, and large-scale clinical trials are still needed. It is a common evidence that CLCNEC harbor driver gene mutations such as EGFR mutation more commonly. The targeted drug has been proven to be clinically effective and can improve OS and achieve higher ORR. However, the available studies regarding CLCNEC treatment are mostly small samples and retrospective, showing discrepant results, contributing to low level of evidence. Hence further research needed to be done to design larger prospective studies to unveil the optimal treatment strategy for CLENEC.

To date, it is still not investigated whether the treatment strategy aiming at LCNEC components ignoring the combination components will lead to acquired-resistant. The constituents of treatment may be selected in accordance with the reported efficacy of the relevant regimens for each component of combined LCNEC. Whether chemotherapy combined with targeted drugs and ICIs could achieve longer OS is a direction for future exploration, which needs a number of clinical trials.
